# Microsatellite marker-based genetic diversity assessment among exotic and native maize inbred lines of Bangladesh

**DOI:** 10.1016/j.sjbs.2023.103715

**Published:** 2023-06-22

**Authors:** Md. Amraul Islam, Md. Shahidul Alam, Md. Maniruzzaman, Muhammad Shahidul Haque

**Affiliations:** a Department of Biotechnology, Bangladesh Agricultural University, Mymensingh, Bangladesh; b On Farm Research Division, Bangladesh Agricultural Research Institute, Mymensingh 2200, Bangladesh; c On Farm Research Division, Bangladesh Agricultural Research Institute, Bagura, Bangladesh; d Bangladesh Wheat and Maize Research Institute (BWMRI), Dinajpur, Bangladesh

**Keywords:** Genetic variability, Inbreds, Polymorphism, SSR markers, *Zea mays*

## Abstract

Hybrid development is basically dependent on the variability among available genetic resources. Polymorphism among the maize inbreds is essentially needed for maize hybridization. This study aimed at the assessment of diversity among 22 maize inbreds by 18 microsatellite markers. The study identified 187 alleles at 18 SSR loci. The amplified allele frequency per microsatellite locus was 10.4 and the highest allele per locus was 17 in SSR primer pair phi026. SSR primer set p-umc1292, phi074 and phi090 showed the lowest 6 alleles per genotype per locus. The locus phi026 showed the highest degree of gene diversity (0.92), and the locus p-umc1292 had the lowest of gene diversity (0.77) with a mean value of 0.862 among the microsatellites. At each site, the most prevalent allele varied between 0.14 (bnlg371) and 0.36. (p-umc1292). At any given locus, an average of 0.22 out of the 22 selected maize inbred lines had a common major allele. The average value of the polymorphic information content (PIC) was 0.85, within the range of 0.74 at the lowest to 0.92 at the highest. The higher PIC values of phi026 and nc013 established them to be the best markers for maize inbred lines. The UPGMA clustering generated seven distinct groups having 12.5% of similarity coefficient. The results revealed that inbred lines E10, E27, E19, E34, E35, E4, E43, E28, E11, E21, E17, E38, E25, E34, E14, E16, E39 and E3 were more diversified. These lines are promising to be used as parent materials for hybrid maize development in the future.

## 1 Introduction

In Bangladesh, maize (*Zea mays* L. subsp. mays) is used as food, feed, and fodder. It is now cultivated worldwide, though it is most suited in wet, and hot climates. It has been observed to flourish in cold, hot, dry, or wet conditions, rendering it a crop suitable for a very diverse climate (Fernandez-Armesto, 2011). Among the world’s cereal crops, maize occupies the 1st position in total production and wheat covers higher acres. In terms of (dry grain) annual production, the global maize production of 1,137 million tons is distinctly more than 50% higher compared to both rice and wheat (757 M t each) which reflects the substantially higher maize grain yields (5.8 tons/ha), mostly linked to wide spread hybrid cultivation and complementing input use (Erenstein et al., 2022). In Bangladesh's rice-based farming system, maize is increasingly becoming important as a cereal crop. Demand of maize in Bangladesh is expanding rapidly, outpacing production increases. The phenomenal rise in area and production of maize was mainly due to the favorable environment for higher productivity and a stable and expanding market as feed for the poultry and livestock. From 2010 to 2019, maize production increased at an average annual rate of 11.40%, with some wheat producers switching to the cultivation of maize (Biswas, 2019).

Diversity in maize at molecular level is very important and has the potential significant role to play in the future breeding program. The genetic diversity study essentially helps understanding the genetic makeup and aids plant breeders to select suitable parental materials for breeding (Al-Badeiry et al., 2014). The genetic diversity study is essential for understanding of the genetic makeup and helps breeders in selecting desirable parents for conducting future breeding (Al-Badeiry et al., 2014). Basically inbred lines serve as a vital resource for genetics and breeding research and are heavily utilized in the development of hybrid maize (Troyer, 2001). Accurate selection of inbred lines from heterotic groups is a prerequisite of efficient utilization of inbred line.

DNA markers estimate genotype variations which are different from morphological and pedigree information, and emerge as more reliable, direct, and useful tools for managing and conserving germplasm. As a result, researchers are adopting molecular markers as valuable tools for genetic variation studies in many crops. Therefore, utilization of DNA markers in breeding, their objectives can be more effectively attained, which eventually leads to a reduction in the number of field trials (Barcaccia, 2009). Isozymes, Random amplified polymorphic DNA (RAPD), Simple sequence repeat (SSR), Inter simple sequence repeat (ISSR), Restriction fragment length polymorphism (RFLP), Amplified fragment length polymorphism (AFLP) and other molecular markers are efficiently employed to measure genetic variability. By the direct genomic examination, these markers demonstrate how similar cultivars are to one another (Almeida et al., 2011). SSRs or microsatellite loci abundant in genomic DNA have been considered sufficient for genetic diversity assessment and increase the breeding efficiency in maize (Rupp et al., 2009, Fernandes et al., 2015). An effort was, therefore, made to estimate the degree of DNA level diversity for choosing diversified inbred lines from 22 genotypes using 18 SSR markers for the development of superior hybrid maize.

## 2 Materials and methods

### 2.1 Inbred maize lines

Seeds of 22 inbred lines collected from various sources and of different countries were used. Out of 22, four inbred lines were obtained from CIMMYT Maxico, eight from Bangladesh and ten from Indian origin. They were developed by continued self-fertilization of cross-pollinated maize species for 6–7 generations to attain near homozygosity. They were maintained by selfing after collection from various sources as mentioned. A detailed description of the origin, source, pedigree and some other important traits of inbred lines used in this study are included in Table 1.

**Table 1:** Inbred code, inbred name, origin and pedigree of maize inbreds investigated under the present study.

**Sl. No.**	**Accession numbers**	**Inbred names**	**Origin**	**Pedigree**
1	E27	E-7	H08R-N8201-37	Pop.31C4S5B-6-#-#-1–2-B-B-B-B-B-B1-B-B-2-B
2	E34	E-95	H08R-N8202-233	P31C4S5B-6-#-#-1–1-B-B-B-B-B-5–4-2-B
3	E25	CML-480	CIMMYT, Mexico	SINTAMTSRC2-88–2-2-B*8
4	E10	IPB-36	Bangladesh	IPB-911–36
5	E4	IPB-22	Bangladesh	IPB-911–22
6	E35	CZ-26	Bangladesh	CZ-2370–26
7	E18	CZ27	Bangladesh	CZ-2370–27
8	E19	E-77	H08R-N8202-177	P31C4S5B-39-#-#-B-B-B-B-B-8–6-3-B
9	E3	CML-285	CIMMYT, Mexico	P24C5F34-2–3-F-2#-BBB-F
10	E20	IPB-16	Bangladesh	IPB-911–16
11	E38	E-67	H08R-N8202-139	P31C4S5B-33-#-#-11-BBBB-B-B-3-B-1-B
12	E6	CLRCY-031	CIMMYT, Mexico	–
13	E36	E-94	H08R-N8202-231	P31C4S5B-6-#-#-1–1-B-B-B-B-B-5–2-2-B
14	E43	CML-192	CIMMYT, Mexico	G34QH174-3–1-2-BB
15	E39	CZ-18	Bangladesh	CZ-2370–18
16	E11	E-101	H08R-N8204-13	CML 421-B-B-B-3-B
17	E14	E-28	H08R-N8202-28	CA03139-7-B-2-B
18	E16	CZ-25	Bangladesh	CZ-2370–25
19	E15	CZ-1	Bangladesh	CZ-2370–1
20	E28	E-104	H08R-N8204-25	CML-474-B-B-B-2-B
21	E21	E-98	H08R-N8204-2	CML469-1-B-B-2-B
22	E17	E-68	H08R-N8202-148	P31C4S5B-33-#-#-11-BBBB-B-B-B-2-B-3-B

### 2.2 DNA extraction

In each inbred line, 10–15 seeds were germinated in pots containing soil under a wire house for plantlet development. Young, active 4–7 folded leaves from 10 to 15 day-old maize seedlings were sampled and were stored in a −20 °C refrigerator until the extraction of DNA. Genomic DNA from a pooled sample of each inbred was extracted using the CTAB method according to Maaß and Klass (1995) with necessary modifications as described afterwards. Maize leaves were ground in a mortar; the powder was extracted in 5% CTAB, 1 M Tris-HCl, pH 8.0, 5 M NaC1, 0.5 M EDTA, 10 mM EGTA, 0.2% 2-mercap-toethanol for 20 min at 60 ∼ in a shaking water bath. A Chloroform: Isoamyl alcohol = 24: 1 (v/v) extraction was performed twice on the suspension, then the DNA was precipitated from the aqueous phase by the addition of 0.6 vol of isopropanol. The DNA was recovered by low-speed centrifugation (10 min at 4000 9), rinsed with 70% and 96% ethanol, dried, and re-dissolved in TE. DNA was purified with propanol following a half-hour treatment with 10 g/ml RNase at 37 °C. In TE buffer, pure DNA was dissolved before being kept at −20 °C. DNA was quantified by measuring the optical density (O.D.) using a Nanodrop Spectrophotometer and DNA quality was also tested by electrophoresis (Haque et al., 2021).

### 2.3 Primers and PCR amplification

Eighteen SSR primers (Table 2) were selected to detect polymorphism based on the strength of the bands, individual constancy, appearance of smearing, and capacity for population discriminating. These primers were previously used and gave the desired performance in relevant previous studies (Hoxha et al., 2004, Kumar et al., 2012, Xia et al., 2000, Yuan et al., 2002). PCR cocktail/master mix after preparation was kept in ice for use. PCR settings were following those reported by Hoxha et al. (2003). The DNA amplification was executed in 18 μl reaction mixture that included 4 µl DNA, 0.25 mM dNTPs, 1.5 mM MgCl_2,_ 5 µl each of the forward primers and reverse primers, 10X PCR buffer (Genei) and 3U Taq DNA polymerase. Then the PCR was run for 35 cycles. Thermal cycles were a preliminary denaturation at 94 °C for 5 min, 30 cycles of 1 min each at 94 °C, annealing for 1 min at 60–65 °C, and for 1.5 min at 72 °C, and the final extension for 10 min at 72 °C. Prior to usage, PCR products were kept at 4 °C.

**Table 2:** Information of microsatellite markers used for the determination of molecular variations.

**Sl. No.**	**Oligo name**	**Sequence of the Primer**	**Chrom^a^ No.**	**Length (bp)**	**Allele size range (bp)**	**Temp^b^ (^0^C)**
1	bnlg371	Forward: ATCTAATCGCAACGCGAAGCAGAGA Reverse: TATCGACCGTAGCTCCGACTGT	2.0	25 22	84–225	72.2 66.9
2	bnlg1014	Forward: CACGCTGTTTCAGACAGGAA Reverse: CGCCTGTGATTGCACTACAC	1.0	24 23	156–170	64.1 64.4
3	bnlg1124	Forward: TCTTCATCTCTCTATCAAACTGACA Reverse: TGGCACATCCACAAGAACAT	1.0	25 20	–	60.9 64.1
4	bnlg1429	Forward: CTCCTCGCAAGGATCTTCAC Reverse: AGCACCGTTTCTCGTGAGAT	1.0	20 20	184–225	63.9 63.8
5	nc013	Forward: AATGGTTTTGAGGATGCAGCGTGG Reverse: CCCCGTGATTCCCTTCAACTTTC	6.04	24 23	102–132	73.3 70.3
6	phi002	Forward: CATGCAATCAATAACGATGGCGAGT Reverse: TTAGCGTAACCCTTCTCCAGTCAGC	1.08	25 25	69–81	71.2 69.1
7	phi026	Forward: TAATTCCTCGCTCCCGGATTCAGC Reverse: GTGCATGAGGGAGCAGCAGGTAGTG	4.04	24 25	78–130	73.3 73.6
8	phi039	Forward: ACCGTGTCTAATGTGTCCATACGG Reverse: CGTTAGGAGCTGGCTAGTCTCA	1.08	24 22	78–129	67.6 64.6
9	phi053	Forward: CTGCCTCTCAGATTCAGAGATTGAC Reverse: AACCCAACGTACTCCGGCAG	3.05	25 20	170–194	66.4 68.2
10	phi069	Forward: AGACACCGCCGTGGTCGTC Reverse: AGTCCGGCTCCACCTCCTTC	5.04	19 20	197–206	71.2 69.0
11	Phi074	Forward: CCCAATTGCAACAACAATCCTTGGCA Reverse: GTGGCTCAGTGATGGCAGAAACT	4.04	26 23	89–95	75.9 68.7
12	Phi090	Forward: CTACCTATCCAAGCGATGGGGA Reverse: CGTGCAAATAATTCCCCGTGGGA	2.08	22 23	141–151	67.9 73.7
13	Phi047	Forward: GGAGATGCTCGCACTGTTCTC Reverse: CTCCACCCTCTTTGACATGGTATG	3.09	21 24	140–152	66.4 67.4
14	p-umc1073	Forward: CACCAACGCCAATTAGCATCC Reverse: GTGGGCGTGTTCTCCTACTACTCA	1.0	21 24	–	68.3 67.3
15	p-umc1292	Forward: GAAGTGGGGAACATGGTTAATGTC Reverse: TCACGGTTCAGACAGATACAGCTC	1.0	24 24	65–68	66.2 66.6
16	p-umc1354	Forward: GATCAGCCCGTTCAGCAAGTT Reverse: GAGTGGAGGCGGAGGATCTG	1.0	21 20	36–62	67.5 68.5
17	p-umc1685	Forward: TAGTTTGAGGGATCAAGAACCACC Reverse: GCTCAAAGGCAAGGCAGTATTTTA	1.0	24 24	–	66.1 66.0
18	p-umc2226	Forward: TGCTGTGCAGTTCTTGCTTCTTAC Reverse: AGCTTCACGCTCTTCTAGACCAAA	1.0	24 24	–	66.5 66.3

### 2.4 Polyacrylamide gel electrophoresis (PAGE) for microsatellite analysis

PAGE is typically used to resolve DNA molecules with low molecular weights between 50 and 100 base pairs (bp) and to identify variations of up to one bp. For the preparation of the PAGE gel, the following chemicals were used: 10xTBE buffer solution, 40% Acrylamide-Bis Acrylamide, 10% Ammonium Persulfate (APS), TEMED, and sterile distilled water (Hannan et al., 2021). Starting from one corner and moving steadily upward, the gel was poured throughout the short plate until the top. The comb was gently inserted to ensure that only half of it was completely immersed in the gel which was left to polymerize for 20 to 25 min. The electrophoresis loading buffer (0.25% bromophenol blue, 30% glycerol, 0.25% xylene cyanol and 1 mM EDTA) was combined with the final reaction result (2 µl). The gel tank was filled with 0.5X TBE and the apparatus was started (Hasanuzzaman et al., 2022). The power supply was turned off once bromophenol blue had covered two-thirds of the gel length. For 90–120 min, electrophoresis was performed at 100 V–400 mA. Ethidium bromides were carefully dissolved in 500 ml of sterile, distilled water to create the staining solution. Two gels were stained using the staining solution. The data were recorded using the UVPRO Alpha Innotech gel documentation equipment after the staining of the gel for a period of 30–35 min in total darkness.

### 2.5 Analysis of microsatellite data

Molecular weights of the products were determined by Alpha-Ease FC 5.0, the binary format data (“1″ and ”0″ indicate the presence and absence of alleles) exported through Power Marker as suggested by Liu and Muse (2005) to analyze using NTSYS-pc (Rohlf, 2002). The following formula was used to generate the polymorphic information content (PIC) values, which are used to measure gene diversity (Anderson et al. 1993):

$$PICi\;=1-\sum_{f=1}^{n}{\left(Pij\right)}^{2}$$

Where, n is the marker alleles for ith marker and P_ij_ is the frequency of the j^th^ allele for ith marker.

Version 3.25 of Power Marker was used as the tool to calculate the summary statistics, including the major allele number, number of alleles per locus, gene diversity, and PIC value following Liu and Muse (2005). A similarity matrix generated using the Simqual subprogram and the Dice coefficient generated a dendrogram outlining the relationships between the genotypes was created using the SAHN subprogram and the Unweighted Pair Group Algorithm with Arithmetic Mean (UPGMA) clustering. The subprograms of the computer software, like D Center, Output, and MXPlot utilized the similarity matrix for principal coordinate analysis (PCA). Numerical Taxonomy computer software and NTSYS-pc D Center, Output, and MXPlot subprograms employed the similarity matrix for PCA.

$$\mathrm{GD}=1-d_{\mathrm{xy}}/\left(d_{x}+d_{y}-d_{\mathrm{xy}}\right)$$

Where, GD = Genetic distance of two genotypes,

d_xy_ = common locus (bands) number in two genotypes,

d_x_ = locus number in genotype 1 and.

d_y_ = locus number in genotype 2.

## 3 Results

**Fig. 1:**
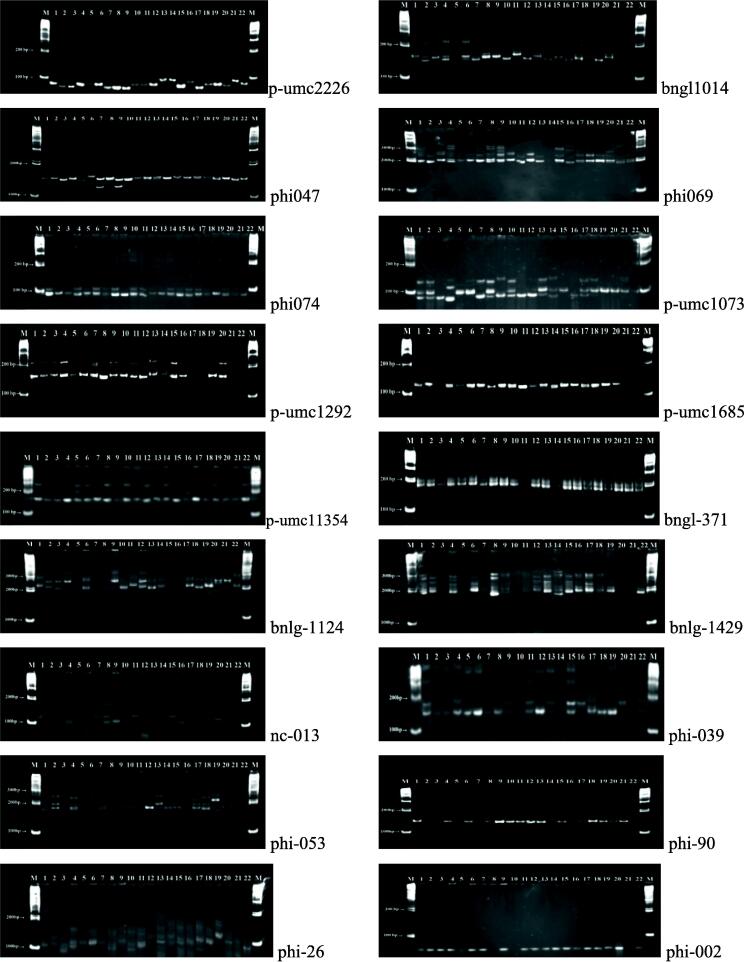
DNA profile of 22 different inbred lines of maize with SSR markers. Accession numbers E27, E34, E25, E10, E04, E35, E18, E19, E03, E20, E38, E06, E36, E43, E39, E11, E14, E16, E15, E28, E21 and E17 were arranged sequentially from 1 to 22, M: Ladder DNA (100 bp).

SSR loci were analyzed for the detection of polymorphism. The patterns of DNA polymorphism detected by 18 SSR markers have been presented in Fig. 1. Showing the average of 10.4 alleles per microsatellite/genotype locus, 187 alleles in total were found at 18 SSR markers among 22 maize inbred lines. SSR primer set phi026, which revealed 17 alleles, displayed most alleles per locus or genotype, followed by nc013 (16 alleles) and bnlg1124 (10 alleles) (15 alleles). SSR primer set p-umc1292, phi074, and phi090 detected the homologous chromosome with the lowest allele number per locus. Those showed a total of 6 alleles per genotype (Table 3). Allele size ranged from 6 to 17 bp. The loci nc013 and phi026 were the most efficient in detecting gene diversity (0.92), and were very closely followed by the loci bnlg1124 (0.91) and bnlg371 (0.90). However, loci p-umc1292 detected the relatively lower gene diversity (0.77). The ultimate mean diversity among the loci was 0.862 (Table 3).

**Table 3:** Number of alleles, range of allele (bp) and gene diversity (GD) found in 22 maize inbred lines for 18 SSR markers.

**Sl.No****.**	**Primer**	**Allele number**	**Gene diversity**	**Allele size (bp)**
1	bnlg371	13	0.90	178–198
2	bnlg1014	12	0.89	145–167
3	bnlg1124	15	0.91	221–321
4	bnlg1429	12	0.89	195–237
5	nc013	16	0.92	100–131
6	phi002	7	0.81	67–72
7	phi026	17	0.92	83–130
8	phi039	9	0.87	146–187
9	phi053	9	0.82	174–217
10	phi069	9	0.87	203–214
11	Phi074	6	0.81	93–97
12	Phi090	6	0.79	140–144
13	Phi047	10	0.86	139–152
14	p-umc1073	12	0.89	84–104
15	p-umc1292	6	0.77	153–162
16	p-umc1354	9	0.87	155–190
17	p-umc1685	9	0.86	118–130
18	p-umc2226	10	0.86	77–97
Total		187	15.51	
Average		10.39	0.86	

The most prevalent alleles at each location had frequencies ranging from 0.14 (bnlg371 and bnlg1124) to 0.36. (p-umc1292). At any given locus, 22 inbred lines of maize had an average of 0.22 significant alleles in common. In this case, every gene frequency below 1 meant that all genes were polymorphic. With values ranging from 0.74 to 0.92, the average PIC was estimated to be 0.85 (Table 4).

**Table 4:** Data on sample size, No. of observation, major alleles frequencies and polymorphism information content (PIC) found from 18 SSR markers among 22 maize inbred lines.

**Sl.No****.**	**Primer**	**No. of observation**	**Major allele size (bp)**	**Major allele frequencies (%)**	**PIC**	**Mean PIC**
1	bngl371	22	178–198	0.14	0.90	0.85
2	p-bnlg1014	22	145–167	0.18	0.88	
3	p-bnlg1124	22	221–321	0.14	0.91	
4	p-bnlg1429	22	195–237	0.18	0.88	
5	nc013	22	100–131	0.14	0.92	
6	phi002	22	67–72	0.27	0.79	
7	phi026	22	83–130	0.18	0.92	
8	phi039	22	146–187	0.18	0.85	
9	phi053	22	174–217	0.32	0.80	
10	phi069	22	203–214	0.18	0.86	
11	Phi074	22	93–97	0.27	0.78	
12	Phi090	22	140–144	0.27	0.76	
13	Phi047	22	139–152	0.23	0.85	
14	p-umc1073	22	84–104	0.18	0.88	
15	p-umc1292	22	153–162	0.36	0.74	
16	p-umc1354	22	155–190	0.18	0.85	
17	p-umc1685	22	118–130	0.23	0.84	
18	p-umc2226	22	77–97	0.27	0.84	

### 3.1 UPGMA similarity matrix

**Fig. 2:**
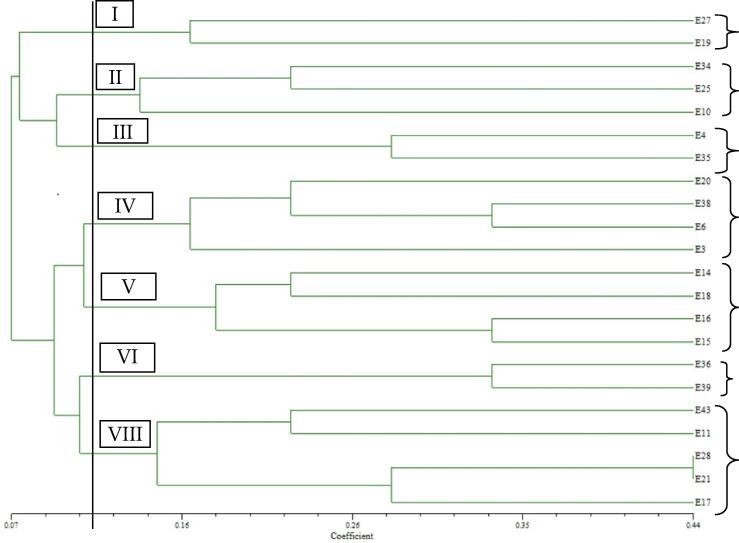
UPGMA dendrogram showing associations among maize inbred lines as revealed by cluster analysis of SSR distance data based on the alleles detected by 18 SSR markers.

The neighbor-joining clustering and the genetic similarity assessment through UPGMA clustering were compatible. Cluster analysis utilizing the UPGMA algorithm and Jaccard's coefficient revealed the genetic link. In addition, the UPGMA clustering algorithm generated seven groups with a similarity coefficient of 12.5% (Fig. 2). Among seven clusters, three clusters formed with two genotypes in each i.e., cluster I (E27 and E19), cluster III (E4 and E35) and cluster VI (E36 and E39). Cluster II accommodated three inbred lines (E34, E25 and E10), cluster IV contains four inbred lines (E20, E38, E6 and E3), Cluster V also contains four inbred lines (E14, E18, E16 and E15) and Cluster VII accommodate the highest number of five inbred lines (E28, E21, E17, E43 and E11) (Fig. 2).

### 3.2 Genetic distance-based analysis

The pair-wise Nei genetic dissimilarity coefficients (Nei, 1973) which was calculated employing 18 SSR markers generated the highest genetic distance (1.00) between inbred E28 and E14, inbred E28 and inbred E19, inbred E39 and inbred E4, inbred E39 and inbred lines E15, inbred lines E39 and E27, inbred lines E16 and E34, inbred lines E16 and E19, inbred lines E38 and E25, inbred lines E38 and E44, inbred E20 and inbred E27, inbred lines E34 and E3 and so on (Table 5).

**Table 5:** Lower and higher inter genotypic distances (Nei, 1973) between pairs of Maize inbred lines based on 18 SSR markers.

	**E03**	**E04**	**E06**	**E10**	**E11**	**E14**	**E15**	**E16**	**E17**	**E18**	**E19**	**E20**	**E21**	**E25**	**E27**	**E28**	**E34**	**E35**	**E36**	**E38**	**E39**	**E43**
**E03**	0.000																					
**E04**	0.889	0.000																				
**E06**	0.944	0.944	0.000																			
**E10**	1.000	1.000	0.833	0.000																		
**E11**	0.778	1.000	0.944	1.000	0.000																	
**E14**	0.889	0.944	0.833	0.889	0.944	0.000																
**E15**	1.000	0.944	0.889	0.889	0.944	0.778	0.000															
**E16**	0.944	0.889	0.833	0.889	0.889	0.833	0.667	0.000														
**E17**	1.000	1.000	0.889	0.889	0.944	0.889	0.778	0.889	0.000													
**E18**	0.889	0.944	1.000	0.889	0.833	0.778	0.833	0.833	1.000	0.000												
**E19**	0.833	1.000	0.944	0.944	0.944	0.889	1.000	1.000	0.944	0.889	0.000											
**E20**	0.778	0.833	0.778	0.944	0.833	0.889	0.833	0.833	0.889	0.833	0.833	0.000										
**E21**	0.944	1.000	0.889	0.833	0.778	1.000	0.944	0.833	0.667	0.944	1.000	0.944	0.000									
**E25**	0.889	0.889	1.000	0.889	0.889	0.944	0.944	0.944	0.944	0.833	1.000	0.778	0.889	0.000								
**E27**	0.889	0.944	0.833	0.833	1.000	0.833	0.889	1.000	0.944	0.889	0.833	1.000	0.944	0.944	0.000							
**E28**	0.944	0.944	0.944	0.944	0.833	1.000	0.833	0.833	0.778	0.889	1.000	0.944	0.556	0.833	0.944	0.000						
**E34**	1.000	0.833	1.000	0.833	0.944	0.944	0.944	0.944	1.000	0.778	0.889	0.889	0.944	0.778	0.833	0.889	0.000					
**E35**	0.889	0.722	0.889	0.944	0.944	1.000	0.944	0.889	0.889	1.000	0.889	0.944	1.000	0.833	1.000	0.944	0.944	0.000				
**E36**	0.944	1.000	0.833	0.944	0.889	0.889	0.944	0.889	0.944	1.000	0.944	0.944	1.000	0.889	1.000	1.000	0.944	0.944	0.000			
**E38**	0.778	0.889	0.667	0.889	0.778	0.944	0.889	0.833	0.889	0.944	0.889	0.778	0.889	1.000	0.944	0.889	1.000	0.944	0.944	0.000		
**E39**	0.833	1.000	0.889	0.944	0.833	0.944	1.000	0.889	0.944	0.889	0.944	1.000	0.833	0.833	1.000	0.722	0.889	0.944	0.667	0.833	0.000	
**E43**	0.944	0.944	0.944	1.000	0.778	0.944	1.000	0.944	0.889	0.944	0.944	0.889	0.833	1.000	1.000	0.833	0.944	1.000	0.944	0.778	0.833	0.000

### 3.3 Principal coordinate analysis (PCoA)

**Fig. 3:**
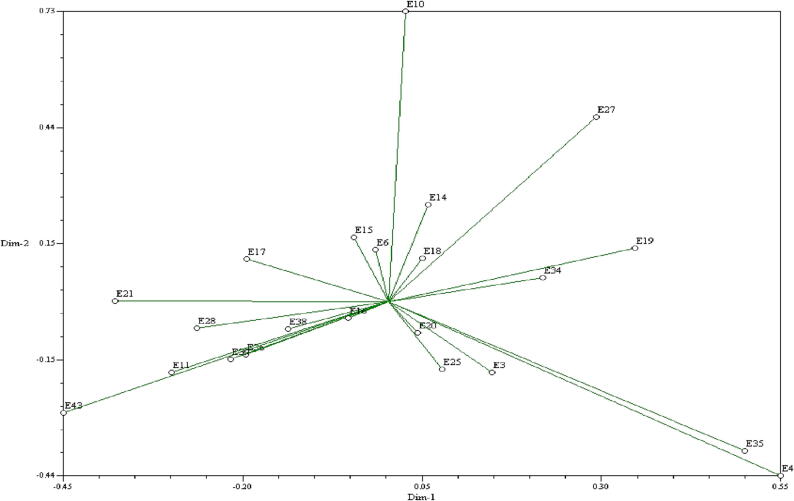
Two-dimensional view of principal coordinate analysis (PCoA) with 18 SSR markers over 22 maize inbred lines.

**Fig. 4:**
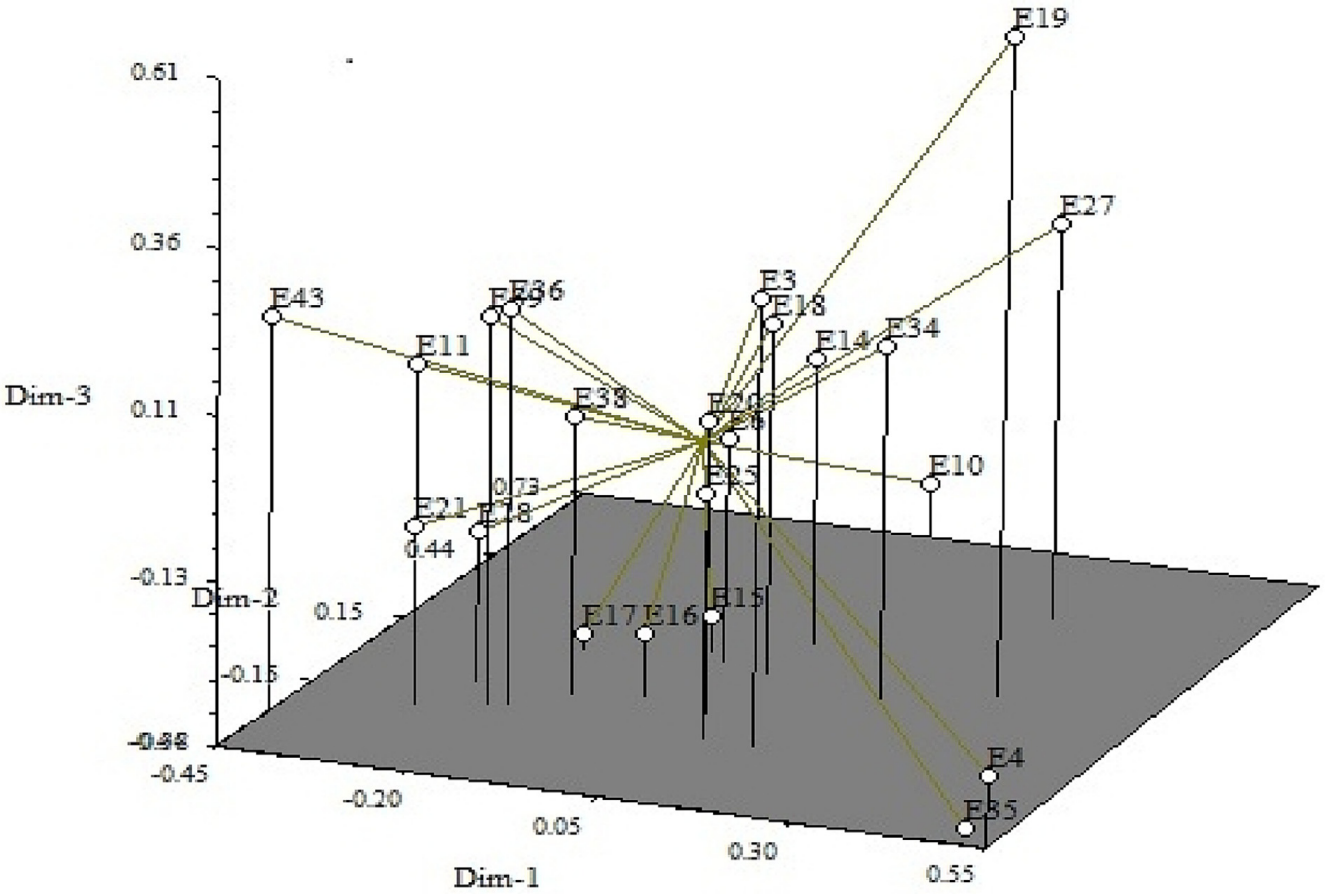
Three-dimensional view of principal coordinate analysis (PCoA) with18 SSR markers over 22 maize inbred lines.

The PCoA in the two dimensional view clearly reflected the spatial arrangement of the maize inbred lines along the principal two axes (Fig. 3). The inbred *viz.* E10, E27, E19, E34, E35, E4, E43, E28, E11, E21, E17, E38, E25, E34, E14, E16, E39 and E3 were distributed quite away from the centroid of the two dimensional graph while the rest of the genotypes were localized around the centroid (Fig. 3). The centroid is the vector that represents the midpoint of the cluster that contains at least one number per variable. The line joining each genotype to the centroid served as a representation of the eigen vectors for that genotype. The arrangement of 22 inbred lines in space along the three main axes was displayed in three dimensions (3D) in the Principal Coordinate Analysis (PCoA) graphical format. The UPGMA cluster analysis result was combined with those of principal coordinate analysis. The three-dimensional picture reinforced the two-dimensional view and made it easier to see four main clusters (Fig. 4).

## 4 Discussion

The advantage of using molecular markers lies in the fact that they are unaffected by environment. Therefore, it is very perfectly feasible to use molecular markers to identify DNA level diversity among maize inbred lines. Breeders can accomplish their objectives more quickly and efficiently by using molecular marker technology, which ultimately reduces the number of field trials for parent selection (Barcaccia, 2009). In the study population, 22 maize inbred lines yielded as many as 187 alleles, giving 10.4 alleles per SSR marker/genotype locus, at 18 SSR markers. SSR primer set phi026 had the highest 17 alleles per locus/genotype. With a total of six alleles per genotype, the SSR primer sets p-umc1292, phi074, and phi090 demonstrated the fewest alleles per locus. This study's average allele count was higher than that of earlier research on maize and wheat genetic diversity (Enoki et al., 2002, Lu and Bernardo, 2001, Vaz Patto et al., 2004, Hasanuzzaman et al., 2022, Hannan et al., 2021, Mathiang et al., 2022). When analyzing 57 CML lines through 85 SSRs, 4.9 alleles per marker were recorded (Warburton et al. 2002). Additionally, Lopes et al. (2015) used 30 microsatellite primers that identified a total of 86 alleles. Pejic et al. (1998) found 6.8 alleles per locus in 33 inbred lines from the United States corn belt employing 27 SSRs, and Xia et al. (2004) found 7.4 alleles in each locus in 155 inbreds using 79 SSRs. Cantagalli et al. (2015) identified 127 alleles from 30 evaluated loci in their genetic diversity and structure study with 31 popcorn accessions using 30 microsatellite primers. In a research involving 260 US inbred lines and 94 SSR loci, Liu et al. (2003) showed that there were on average 21.7 alleles per locus. In diversity studies, the number of alleles detected is often inversely proportional to the corresponding sample size, and the differences observed might be attributed to different sampling techniques. However, another element that affects the frequency of alleles is the use of di-nucleotide repeat SSRs that could also produce a higher frequency of alleles. Furthermore, the higher allelic richness that was discovered may perhaps be attributed to the more diverse group of inbred from the study's gene pool collection. With a mean diversity of 0.862, loci p-umc1292 had the lowest gene diversity (0.77) and loci phi026 gave greatest gene diversity value (0.92). It was shown that the markers detecting fewer alleles had less gene diversity in comparison to those detecting more alleles. The findings of this research are in line with earlier studies by Herrera et al. (2008). At each locus, the most prevalent allele's frequency ranged from 0.14 (bnlg371) to 0.36. (p-umc1292). At any given locus, 22 inbred lines of maize had an average of 0.22 significant alleles in common. The results of the current investigation were consistent with those previously published by Salami et al. (2016) and Lopes et al. (2015).

With a mean of 0.85, the PIC values ranged from 0.74 to 0.92. PIC values revealed that phi026 and nc013 were the most efficient in detecting the polymorphic information content in 22 selective inbreds of maize followed by p-bnlg1124. Lopes et al. (2015) corroborated the present result with their findings that the PIC ranged from 0.19 (Umc2319) to 0.71 (Umc2205). Six loci were very informative (PIC = 0.5); only two loci were uninformative (PIC = 0.25) whereas the remaining 22 loci were moderately formative (0.25 PIC 0.50). Additionally, the average PIC values of this investigation and previously reported results in inbred lines of maize using SSR markers were in good agreement (Heckenberger et al., 2002, Vaz Patto et al., 2004, Lopes et al., 2015). Based on their genetic connections, PIC determines the information of the SSR loci including their capacity to detect changes among the inbreds. Salami et al. (2016) obtained a total of 227 polymorphic bands and demonstrated a high level of genetic variety (Shannon index = 0.51), which further supported our finding. PIC at the SSR loci ranged from 0.58 to 0.81, with the mean value of 0.71.

The neighbor-joining clustering and the genetic diversity analysis by UPGMA were compatible. Genetic links were determined by UPGMA analysis and Jaccard's coefficient. UPGMA analysis further generated seven clusters with a similarity coefficient of 12.5%. Three clusters (I, III, and VI) formed with two genotypes out of the seven clusters.. Cluster II accommodate three inbred lines, cluster IV it contains four inbred lines, Cluster V also contains four inbred lines and Cluster VII accommodate the highest number of five inbred. Salami et al. (2016) grouped 187 maize accessions into four clusters in each area. Lopes et al. (2015) developed seven genetically different groups of maize inbred which fully supported our present study.

The majority of the inbred pairings have the highest genetic distance values, depending on the 18 SSR marker-based pair-wise genetic dissimilarity coefficients. So, there is enough opportunity for the development of superior hybrids. These results revealed that these genotypes were divergent and concurred with those of the Principal Coordinate Analysis. In comparison to genotypes located close to the centroid, those that were put further from the centroid were more genetically divergent. Centroid, on the other hand, can be described as the vector corresponding to the center of the cluster that contains at least one value for each variable. The line joining each genotype to the centroid served as a picture of its eigen vector. The 22 maize inbred lines were spatially distributed along the three main axes in the 3D graphical view of PCoA. The outcome from the UPGMA cluster analysis worked well with the PCoA. The three-dimensional diagram supported a two-dimensional view and made it easier to see four main groupings. Cantagalli et al., 2015, Lopes et al., 2015, and Salami *et al.* (2015) all confirm this finding. The inbred lines with the highest genetic diversity are scattered throughout several clusters, and because they exhibit greater genetic diversity, they are positioned far from the centroid of the two-dimensional PCoA graph.

## 5 Conclusion

Our findings substantiate the availability of significant genetic variations among all the inbred lines, SSR markers clearly grouping the 22 inbred lines into seven clusters at a similarity coefficient of 12.5%. The pair-wise genetic dissimilarity coefficients calculated on the basis of 18 SSR markers show that the bulk of the inbred pairs had maximal genetic distance values. The two-dimensional graph's centroid was located far from the other genotypes, where the remaining 18 inbred lines were distributed. Therefore, there is considerable scope for the development of superior hybrids using the knowledge obtained from this study. Breeders can use these results to select parents for maize breeding program crossing combinations.

## Declaration of Competing Interest

The authors declare that they have no known competing financial interests or personal relationships that could have appeared to influence the work reported in this paper.

## References

[b0005] Al-Badeiry N.A.H., Al-Saadi A.H., Merza T.K. (2014). Analysis of genetic diversity in maize (*Zea mays* L.) varieties using simple sequence repeat (SSR) Markers. J. Babylon Univ..

[b0010] Almeida C., Amorim E.P., Barbosa-Neto J.F., Cardoso-Filho J.A., Sereno M.J.C.M. (2011). Genetic variability in populations of sweet corn, common corn and teosinte. Crop Breed. Appl. Biotechnol..

[b0015] Anderson J.A., Churchil G.A., Autrique J.E., Tanksley S.O., Sorrels M.E. (1993). Optimizing parent selection for genetic linkage maps. Genome.

[b0020] Barcaccia G., Mohan J.S., Brar D.S. (2009). Molecular Techniques in Crop Improvement. 2ed.

[b0025] Biswas J.K., Chowdhury A.M.R., Husain M., Saleque M.A., Brammer H. (2019). From the Ground Up: BRAC’s innovations in the Development of Agriculture in Bangladesh and Beyond.

[b0030] Cantagalli, L.B,, Saavedra, J., Lopes, A.D., Mangolin, C.A., Machado, M.D.F.P.D.S., Scapim, C.A., 2015. Population structure and genetic diversity of Brazilian popcorn germplasm inferred by microsatellite markers. Electronic Journal of Biotechnology. 18, 181–187.

[b0035] Enoki H., Sato H., Koinuma K. (2002). SSR analysis of genetic diversity among maize inbred lines adapted to cold regions of Japan. Theory Appl. Genet..

[b0040] Erenstein O., Jaleta M., Sonder K. (2022). Global maize production, consumption and trade: trends and R&D implications. Food Sec..

[b0045] Fernandes E.H., Schuster I., Scapim C.A., Vieira E.S.N., Coan M.D. (2015). Genetic diversity in elite inbred lines of maize and its association with heterosis. Genet. Mol. Res**.**.

[b0050] Fernandez-Armesto F. (2011).

[b0055] Hannan M.A., Saha N.R., Roy S.K., Woo S., Haque M.S. (2021). Genetic diversity analysis and molecular screening for salinity tolerance in wheat germplasm. Plant Breed. Biotech..

[b0060] Haque, M.S., Saha, N.R., Islam, M.T., Islam, M.M., Kwon, Soo, Jeong, Roy, S.K., Woo, Sun, Hee. 2021. Screening for drought tolerance in wheat genotypes by morphological and SSR markers. J. Crop Sci. Biotechnol. 24. 27–39 https://doi.org/10.1007/s12892-020-00036-7

[b0065] Hasanuzzaman, Saha, N.R., Farabi, S., Tahjib-Ul-Arif, M., Yasmin, S., Haque, M.S., 2022. Screening of salt-tolerant wheat (Triticum aestivum L.) through morphological and molecular markers. Cereal Research Communications, DOI: 10.1007/s42976-022-00278-x

[b0070] Heckenberger M.A., Melchinger E., Ziegle J.S., Joe L.K., Hauser J.D., Hutton M., Bohn M. (2002). Variation of DNA fingerprints among accessions within maize inbred lines with regard to the identification of essentially derived varieties. Genetic and technical sources of variation in SSR data. Mol. Breed..

[b0075] Herrera T.G., Duque D.P., Almeida I.P., Nunez G.T., Alejandro J.P., Martinez C.P., Tohme J.M. (2008). Assessment of genetic diversity in Venezuelan rice cultivars using simple sequence repeats markers. Electron. J. Biotechnol..

[b0080] Hoxha S., Shariflou M.R., Sharp P. (2003). Evaluation of genetic diversity in Albanian maize using SSR markers. Maydica.

[b0085] Hoxha S., Shariflou M.R., Sharp P. (2004). Evaluation of genetic diversity in Albanian maize using SSR markers. Maydica.

[b0090] Kumar A., Rakshit A., Mangilipelli N.K., Varalaxmi Y., Vijayalakshmi T., Vanaja J.M., Yadav S.K., Venkateswarlu B., Maheswari M. (2012). Genetic diversity of maize genotypes on the basis of morpho-physiological and simple sequence repeat (SSR) markers. Afr. J. Biotechnol..

[b0095] Liu K., Goodman M., Muse S., Smith J.S., Buckler E., Doebley J. (2003). Genetic Structure and Diversity among Maize Inbred Lines as Inferred From DNA Microsatellites. Genetics.

[b0100] Liu K., Muse S.V. (2005). Power Marker: an integrated analysis environment for genetic marker analysis. Bioinformatics.

[b0105] Lopes, A.D., Scapim, C.A., Machado, M.D.F.P.D.S., Mangolin, C.A., Silva, T.A., Cantagali, L.B., Teixeira, F.F., Mora, F., 2015. Genetic diversity assessed by microsatellite markers in sweet corn cultivars. Science of Agriculture. 72, 513-519.

[b0110] Lu H., Bernardo R. (2001). Molecular marker diversity among current and historical maize inbreds. Theor. Appl. Genet..

[b0115] Maaß H.I., Klass M. (1995). Infraspecific differentiation of garlic (*Allium sativum* L.) by isozyme and RAPD markers. Theor. Appl. Genet.**.**.

[b0120] Mathiang E.A., Sa K.J., Park H., Kim Y.J., Lee J.K. (2022). Genetic diversity and population structure of normal maize germplasm collected in South Sudan revealed by SSR markers. Plants.

[b0125] Nei M. (1973). Genetic distance between populations. Am. Nat.**.**.

[b0130] Pejic I., Ajmone-Marsan P., Morgante M., Kozumplick V., Castiglioni P., Taramino G., Motto M. (1998). Comparative analysis of genetic similarity among maize inbred lines detected by RFLPs, RAPDs, SSRs and AFLPs. Theor. Appl. Genet..

[b0135] Rohlf, F., 2002. NTSYS-pc: Numarical taxonomy and multivariate analysis system, 2.2 Edn. Department of Ecology and Evolution, State University of NY, Stony Brook.

[b0140] Rupp J.V., Mangolin C.A., Scapim C.A., Machado M.F.P.S. (2009). Genetic structure and diversity among sweet corn (su1-germplasm) progenies using SSR markers. Maydica.

[b0145] Salami H., Sika K., Padonou W., Aly D., Yallou C., Adjanohoun A., Kotchoni S., Baba-Moussa L. (2016). Genetic diversity of maize accessions (*Zea mays* L.) cultivated from benin using microsatellites markers. Am. J. Mol. Biol**.**.

[b0150] Troyer A.F. (2001).

[b0155] Vaz, Patto, M.C., Šatoviæ, Z., Pego, S., Fevereiro, P., 2004: Assessing the genetic diversity of Portuguese maize germplasm using microsatellite markers. Euphytica. 137, 63-72.

[b0165] Warburton M.L., Zianchun X., Crossa J., Franco J., Melchinger A.E., Frisch M., Bohn M., Hoisington D. (2002). Genetic characterization of CIMMYT inbred maize lines and open pollinated populations using large scale fingerprinting methods. Crop Sci..

[b0170] Xia, X.C., REIF, J.C., Zoisington, D.A., Melchinger, A.E., Frisch, M., Warburton, M.L.. 2004. Genetic diversity among CIMMYT maize inbred lines investigated with SSR markers. Low land tropical maize. Crop Science. 44, 2230-2237.

[b0175] Xia X.C., Warburton M.L., Hoisington D.A., Bohn M., Frisch M., Melchinger A.E. (2000).

[b0180] Yuan, L., Zhang,S., Warburton, M., Li, X., Fu, J., Li, M., 2002. Assessment of genetic similarities among maize inbred lines using SSR markers. In Proceedings of the Eighth Asian Regional Maize Workshop, Bangkok. pp. 50-58.

